# Iberian Peninsula and Balearic Island Bathynellacea (Crustacea, Syncarida) database

**DOI:** 10.3897/zookeys.386.6296

**Published:** 2014-03-06

**Authors:** Ana I. Camacho, Beatriz A. Dorda, Isabel Rey

**Affiliations:** 1 Museo Nacional de Ciencias Naturales, CSIC, c/José Gutiérrez Abascal 2, 28006, Madrid, Spain

**Keywords:** Crustacea, Malacostraca, Syncarida, Bathynellacea, Parabathynellidae, Bathynellidae, Invertebrates collections, groundwater fauna, Iberian Peninsula, Balearic Islands

## Abstract

This is the first published database of Bathynellacea. It includes all data of bathynellids (Crustacea, Bathynellacea) collected in the last 64 years (1949 to 2013) on the Iberian Peninsula and Balearic Island. The samples come from groundwater (caves, springs, wells and hyporrheic habitat associated rivers) from both sampling campaigns and occasional sampling conducted throughout the Iberian Peninsula and Balearic Islands. The dataset lists occurrence data of bathynellids distribution, sampling sites (with localities, county and geographic coordinates), taxonomic information (from family to species level) and sampling sources (collector and sampling dates) for all records. The descriptions of new species and species identifications have been carried out by an expert taxonomist (AIC) with 25 years experience in the bathynellids studies (see references). Many of the sampling sites are type localities of endemic species from Iberian Peninsula. The dataset includes 409 samples record corresponding to two families, 12 genera and 58 species, 42 of them formally described plus 16 taxa unpublished and 47 samples in study. All species known from the study area are included, which nearly sum up a quarter of species of Bathynellacea known in the world (250 species).

## General description

**Figure 1.**
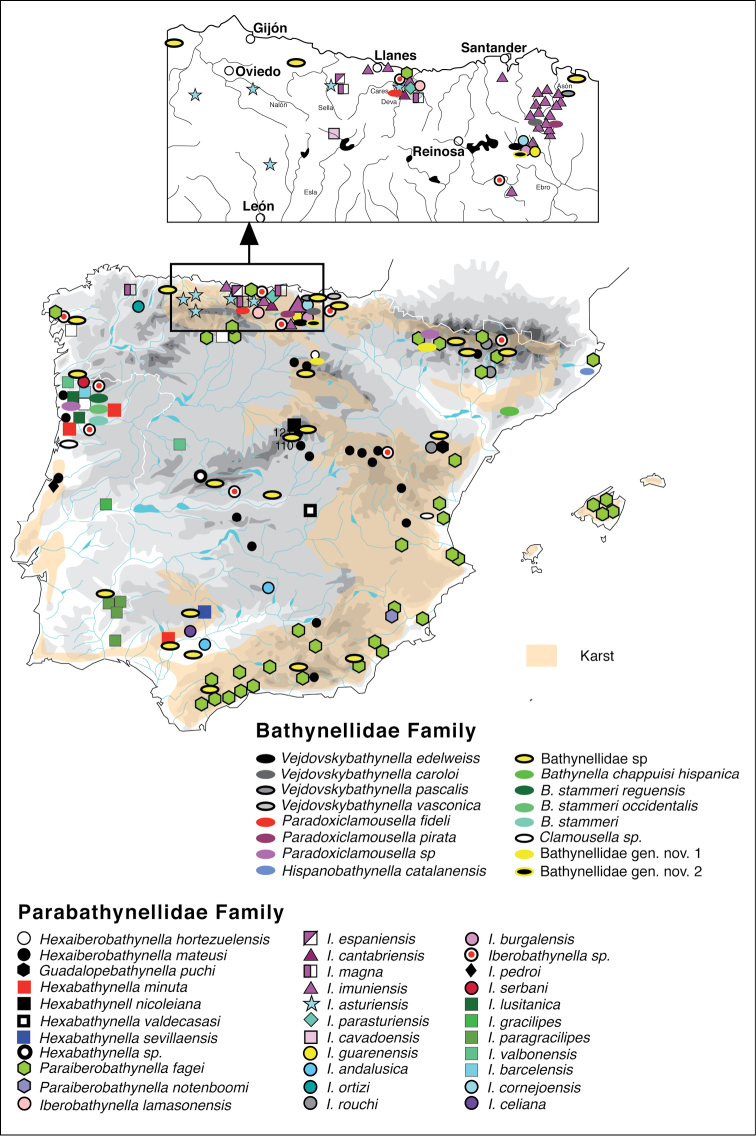
Distribution map of Syncarida Bathynellacea records from Iberian Peninsula and Balearic Islands.

**Purpose:** The Iberian Peninsula and Balearic Islands is currently one of the best-studied regions in terms of bathynells and their distribution, and it is also the region with the highest diversity of this group of crustacean in the world (Camacho 1987b, 1988a, b, 1989c, 1994, 1998, 2000, 2003a, b, 2004, 2005a, b, 2006; Camacho and Coineau 1989; Camacho and Puch 2008; Camacho and Serban 2000; Camacho and Valdecasas 2003, 2008; Camacho et al. 1997, 2000, 2006, 2011, 2012, 2013a; Deharveng et al. 2009; Malard et al. 2009). Nevertheless, until now, no single complete dataset compiling all this information had been published and made available to the public. Within the study region considered there are still many sites to be explored from the biospeleology point of view, but the volume of information already available recommends compiling it and making it accessible to the public. Due to the fact that Syncarida Bathynellacea are animals that live exclusively in subterranean waters (stygobionts), which are difficult to access for man, and that their taxonomic study is complex, the knowledge we have of this group within the framework of global biodiversity is scarce (Camacho and Valdecasas 2008), even though they are an important element of the groundwater fauna (Camacho et al. 2012). Their habitat is seldom sampled, and their presence and density in the samples taken are normally low, which is the reason why this group is considered rare and with low diversity. Currently there are 250 species described worldwide, and most of them are only known from their type locality, or from a small area around it (Camacho et al. 2012). Considering that within our region of study more than 58 different species are known (42 formally described, 16 new species and 47 samples being studied, see Table 1) along a large part of the territory included (see Fig. 1) – this represents almost a quarter of all the species known worldwide – we believe it is time to compile all the information generated in the last 30 years to make it available to the scientific community. The purpose of this paper is to document a dataset corresponding to 409 records of Bathynellacea, from 195 localities (some of them sampled on several occasions) where we have identified the presence of 2 families, 12 genera (2 pending publication) and 58 species (16 pending publication), plus a number of samples currently identified only to family level. Most of the information comes from our own sampling and taxonomic identification (AIC), although a few refer to bibliographic data (only 16). This last information refers to the type locality of 12 species and 4 subspecies and their original description. These are mostly samples from Portugal, although the species have later been found in other localities too. Also, the first identification of the specimen *Paraiberobathynella* cf. *fagei* from a Spanish locality is part of this set of records (this species was later found in other localities too). Some of this information has never been published, and other can be found but in separate sources distributed along an extended period of time, so we deemed it necessary to pool all information into a single dataset containing all the information available for each sample of bathynell. This way, the dataset is a significant contribution of basic information on Iberian Bathynellacea, which due to the rareness of the species and their extreme habitat can be useful for subterranearn biodiversity, ecology and conservation studies, as well as for Global Change estimations (the dataset includes sampling efforts in successive years). Our aims for publishing this dataset are 1) providing information on the diversity and distribution of the Iberian and Macaronesic groundwater fauna, 2) describing the bathynellacea collection of AIC and the MNCN, and 3) offering the first dataset of bathynellacea in the World to the scientific community in the hopes of promoting other researchers to publish their groundwater fauna datasets.

**Table 1.** Number of species within each of the genera present in the Iberian Peninsula and Balearic islands, and number of samples in the study.

Genera	Published species	Cryptic and unpublished new species	Sampled in study of Bathynellacea
*Iberobathynella*	22	5	14
*Paraiberobathynella*	2	0	0
*Hexaiberobathynella*	2	0	0
*Guadalopebathynella*	1	0	0
*Hexabathynella*	4	1	0
*Vejdovskybathynella*	4	3	0
*Paradoxiclamousella*	2	2	0
*Clamousella*	0	3	0
Bathynellidae gen. n. 1	0	1	0
*Hispanobathynella*	1	0	0
*Bathynella*?	4	0	33
Bathynellidae gen. n. 2	0	1	0
**TOTAL**	**42**	**16**	**47**

**Additional information:** Section 2 of the bibliography includes a list of the publications citing the bathynells included in this dataset. Table 3 includes information on all the new species of Bathynellacea described since 1986 until the present, including the catalogue number of the type series in the classic Crustacea collection of the MNCN, as well as the vouchers of the Tissue and DNA Collection of the MNCN referring to the DNA extractions from specimens of type localities where available.

## Project details

**Project title:** Database all records the Bathynellacea in the Iberian Peninsula and Balearic Island

**Personnel digitisation:** Camacho AI and Dorda BA

**Determination specialist:** Camacho AI

**Administrative contact:** Dorda BA

**Bathynellacea determination specialist:** Camacho AI

**Funding:** Fauna Ibérica I (DGICYT PB87-0397); Fauna Ibérica II (DGICYT PB89-0081); Fauna Iberica III (DGICYT PB92-0089); Inferencia de Patrones Biogeográficos a pequeña escala (DGICYT PB96-0894); Inventario y Catalogación informática de la Biodiversidad acuática subterránea de la Península Ibérica, Baleares y Macaronesia (CICYT REN2000-2004 GLO); Protocols for the Assessment and Conservation of aquatic life in the subsurface (PASCALIS), European Union Proposal EVK2-2001-00086 (Contract: EVK2-CT-2001-00121); Biodiversidad Faunística en el sector turístico del Complejo Ojo Guareña: Evaluación de la Influencia de la presión humana en algunas de sus poblaciones de invertebrados (Contract CSIC- Junta de Castilla León, 2002-2004); Sobre el origen y distribución de la fauna acuática subterránea (CICYT CGL2005-02217/BOS); Colonización, Éxito Evolutivo y Biodiversidad Faunística del Complejo Kárstico de Ojo Guareña” En el Monumento Natural de Ojo Guareña (Burgos) (Contract CSIC- Junta de Castilla León, 2006-2009); Estudio piloto para la detección a diferentes escalas geográficas de procesos evolutivos relacionados con el origen de la biodiversidad en grupos de invertebrados singulares (MICINN CGL2010-15786, subprograma BOS).

**Study area descriptions/descriptor:** The study area includes 195 sites throughout the Iberian Peninsula and Balearic Island, and several sampling dates ranging from 1949 to 2013.

Most localities sampled are in karstic areas (Ayala Carcedo et al. 1986; García-Codrón 1983; Puch 1998). Sampling is always done in groundwater caves, springs, wells and interstitial environment of the epigen river where the stygobionts living in them can be collected. The general aim, apart from the specific objectives of each project, was identifying the Bathynellacea crustacean fauna inhabiting subterranean waters of Spain and Portugal (Fauna Ibérica).

**Design description:** This dataset was developed to determine the current distribution patterns of bathynellids species at the scale of the Iberian Peninsula. It also contributes to the knowledge of groundwater Biodiversity in the Iberian Peninsula and to identify endemic fauna at different geographic scales (country, counties and localities). Prior to digitisation, the taxonomic identification pre-existing was reviewed by the specialist AIC. The dataset is exported to DarwinCore v1.2 format and uploaded to the IPT of the GBIF Spanish node (http://www.gbif.es:8080/ipt). DarwinCore elements included in the dataset structure are listed in the dataset description section.

### Data published through

GBIF: http://www.gbif.es:8080/ipt/resource.do?r=mncn-aic

## Taxonomic coverage

**Figure 2.**
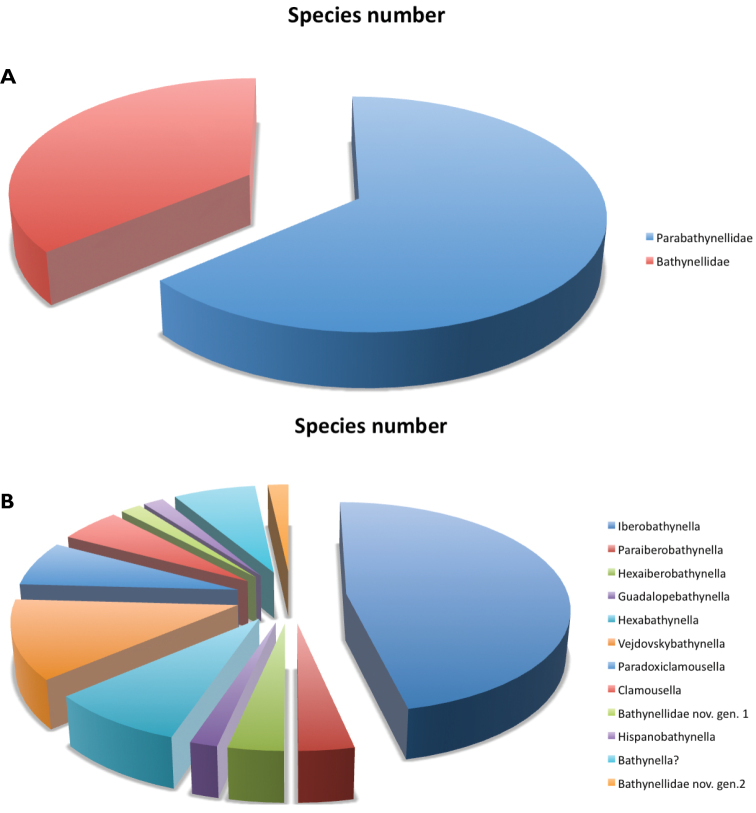
Distribution of Syncarida Bathynellacea from Iberian Peninsula and Balearic Islands. **A** species within families and **B** species within genera of Parabathynellidae and Bathynellidae families.

**General taxonomic coverage description:** This is a collection of Bathynellacea, a group of Crustacea Malacostraca, contains all known species for Spain and Portugal as well as all the localities where bathynells have been found within the region considered. The collection includes all the material obtained in the Iberian Peninsula and Balearic Islands except the samples collected between 1949 and 1968 in Portugal, which have been lost. Most of the collection is identified to species level. The samples without identification to species level, due to the lack adult specimens or the absence of males, have been identified to genus or family level. We have found 12 genera belonging to two families (Table 1), Parabathynellidae (63,8% of the species and 68% of the records) and Bathynellidae (36,2% of the species and 32% of the records) (Figure 2A). In the Parabathynellidae family five genera have been identified: *Iberobathynella* Schminke, 1973 (22 species plus five unpublished found in all habitat), *Paraiberobathynella* Camacho & Serban, 1998 (two species, found in wells and interstitial river bank), *Hexaiberobathynella* Camacho & Serban, 1998 (two species found in wells and interstitial river bank), *Guadalopebathynella* Camacho & Serban, 1998 (one species found in interstitial river bank) and *Hexabathynella* Schminke, 1972 (four species plus one unpublished found in caves and interstitial river bank) (see Figure 2B). In the Bathynellidae family seven genera have been identified: *Vejdovskybathynella* Serban & Leclerc, 1984 (four species plus three unpublished found only in caves), *Paradoxiclamousella* Camacho et al., 2013 (two species plus two unpublished found in caves, spring and interstitial river bank), *Clamousella* Serban, Coineau & Delamare Deboutteville, 1971 (three unpublished species found in interstitial river bank), *Hispanobathynella* Serban, 1989 (one species in a cave), *Bathynella* Vejdovsky, 1882 (cf) (four species incerta sedis found in interstitial river bank and one cave), Bathynellidae gen. nov. 1 (genus and species unpublished found in wells and interstitial river bank) and Bathynellidae gen. nov. 2 (genus and species unpublished found in a cave) (Figure 2B). In addition there are 47 sample more, 33 of the Bathynellidae family and 14 of the Parabathynellidae family, still in study and probably a number of them belonging to new genera. In summary, until now we have identified 58 species (16 unpublished), all endemic from Portugal and Spain. Twenty seven of these, have been described as new species only in recent years (see Table 3 and Reference List 2). The other 16 species still pending formal description, are also new to science. This dataset includes all species of Bathynellacea known for the study area, and nearly a quarter of all the species known worldwide (Camacho 2006, Camacho and Valdecasas 2008).

## Taxonomic ranks

**Kingdom:** Animalia

**Phylum:** Arthropoda

**Class:** Crustacea

**Order:** Bathynellacea

**Family:** Bathynellidae, Parabathynellidae.

**Common names:** doesn’t exist

## Spatial coverage

**Figure 3.**
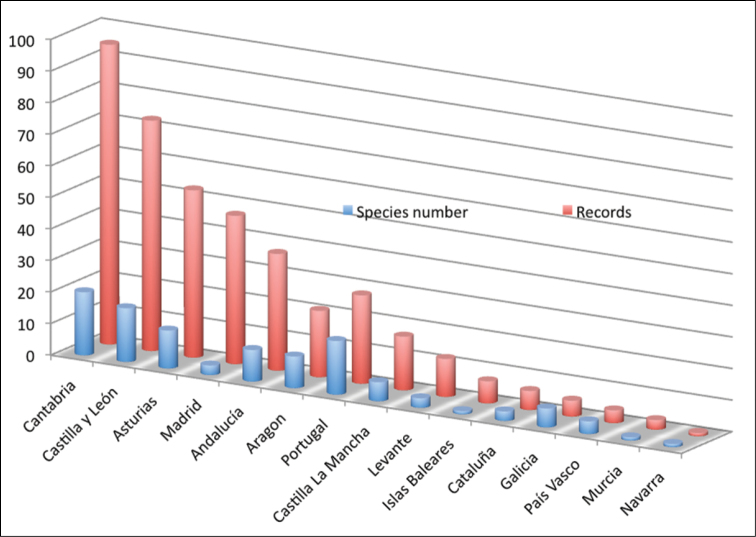
Distribution of records in the dataset by regions (Autonomous Communities).

**Figure 4.**
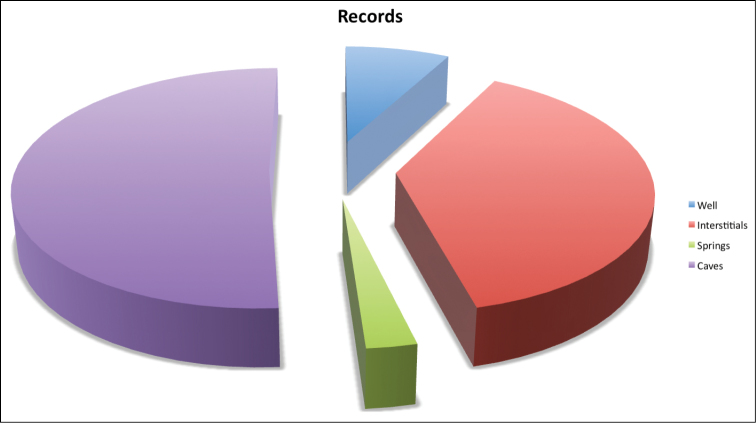
Distribution of records in the dataset by habitat sampled.

**General spatial coverage:** The study area includes 195 sites throughout the Iberian Peninsula and Balearic Island (Figure 1). Most of the samples come from Spain (93,1%) and only a small portion (3,9%) from Portugal (with 17 species registered) (Table 2 and Figure 3). The region with most samples and most species is Cantabria (23.2% of records and 20 species) followed by Burgos (13.4% of the records and 11 species) and Asturias (11.7% of the records and 12 species); from all of Andalucía there are 37 records in total (9%) and 21 records from Aragón (5.1%) followed by only 4.2% of the records from Levante. In other provinces included in the dataset there less than 3 records (Salamanca, Pontevedra, La Coruña, Álava, Lugo, Cuenca, Navarra, León, Gerona, Lérida and Vizcaya), while from Madrid there are 47 records but these come from only 3 localities sampled many times showing only 3 different species. Regarding the Balearic Islands, only 7 records are included (samples from caves) from the island of Mallorca (1.7% of the records and only 1 species) (Figure 3). There are no records from the provinces of Zamora, Barcelona, Cáceres, Badajoz, Albacete, Segovia, Guipúzcoa and Logroño. Considering the habitats sampled, most of them come from caves (51,1%), mainly from Cantabria (40.7%); interstitial epigean river banks (38.4%), mainly from Madrid and Portugal; a few records are from springs (2.7%), mainly from Cantabria; and 32 records are from wells found mainly in Andalucía and Levante (see Table 2). The sample distribution by provinces and habitat can be seen in Figures 3 and 4 respectively.

**Table 2.** Distribution of records by habitats and Spanish provinces (and Portugal) in the area of study, and total number of species identified in each zone.

	Wells samples	Interstitials samples	Springs samples	Caves samples	Total samples number	Identified species number
Portugal	3	25	0	0	**28**	17
Huelva	5	0	0	0	**5**	2
Salamanca	0	1	0	0	**1**	1
Cantabria	0	5	5	85	**95**	20
Asturias	0	3	2	48	**53**	12
Burgos	0	1	2	55	**58**	11
Sevilla	1	6	0	2	**9**	6
León	0	4	0	1	**5**	3
Pontevedra	0	1	0	0	**1**	1
Soria	6	1	0	0	**7**	4
Huesca	0	13	0	2	**15**	8
Álava	0	0	1	2	**3**	3
Teruel	0	6	0	0	**6**	6
Lugo	0	0	0	1	**1**	1
Córdoba	0	2	0	0	**2**	2
La Coruña	0	1	0	0	**1**	1
Toledo	0	9	0	0	**9**	4
Málaga	3	5	0	1	**9**	3
Cuenca	0	1	0	0	**1**	1
Mallorca	0	0	0	7	**7**	1
Tarragona	0	0	0	2	**2**	1
Murcia	2	0	0	1	**3**	1
Alicante	7	0	0	0	**7**	2
Valencia	1	2	0	0	**3**	2
Almería	2	0	0	0	**2**	2
Cádiz	1	1	0	0	**2**	1
Navarra	1	0	0	0	**1**	1
Gerona	0	2	0	0	**2**	2
Castellón	0	2	0	0	**2**	2
Jaén	0	1	1	0	**2**	2
Granada	0	6	0	0	**6**	3
Orense	0	2	0	0	**2**	2
Lérida	0	2	0	0	**2**	2
Madrid	0	46	0	1	**47**	3
Guadalajara	0	7	0	0	**7**	1
Ávila	0	2	0	0	**2**	2
Vizcaya	0	0	0	1	**1**	1
**Total**	**32**	**157**	**11**	**209**	**409**	**–**

**Table 3.** New species of Crustacea Syncarida Bathynellacea from Spain described since 1980 until present day that are included in the DB of AIC, with the voucher number of type material deposited in the collections of the MNCN (Crustacea and Tissues and DNA). *** Type material in l’Institut de Spéologie “É. Racovitza”, Bucurest (Roumania).

New taxa	Author	Year	Reference	Voucher Number Type material MNCN Collections
Parabathynellidae				
***Iberobathynella***	Schminke	1973		
*Iberobathynella andalusica*	Camacho	2007	Graellsia 63(2): 339-347	20.04/7966-7967 & ADN/29418
*Iberobathynella burgalensis*	Camacho	2005	Graellsia 61(1): 123-133	20.04/6063 & ADN/29520-29521
*Iberobathynella lamasonensis*	Camacho	2005	Journal of Natural History 39(21): 1819-1838	4/20/5911
*Iberobathynella cornejoensis*	Camacho	2005	Journal of Natural History 39(21): 1819-1838	4/20/5912
*Iberobathynella serbani*	Camacho	2003	Journal of Natural History 37: 2885-2907	4/20/5321
*Iberobathynella celiana*	Camacho	2003	Journal of Natural History 37: 2885-2907	20.04/5323 & ADN/29452
*Iberobathynella guarenensis*	Camacho	2003	Journal of Natural History 37: 2885-2907	4/20/5322
*Iberobathynella pedroi*	Camacho	2003	Journal of Natural History 37: 2885-2907	4/20/5320
*Iberobathynella cantabriensis*	Camacho et Serban	1998	Travaux de l’Institut de Spéologie “É. Racovitza” 34: 15-75	4/20/4639
*Iberobathynella paragracilipes*	Camacho et Serban	1998	Travaux de l’Institut de Spéologie “É. Racovitza” 34: 15-75	4/20/4638
*Iberobathynella magna*	Camacho et Serban	1998	Travaux de l’Institut de Spéologie “É. Racovitza” 34: 15-75	***
*Iberobathynella parasturiensis*	Camacho et Serban	1998	Travaux de l’Institut de Spéologie “É. Racovitza” 34: 15-75	20.04/4640 & ADN/29556, 29561, 29566 & 29583
*Iberobathynella ortizi*	Camacho	1989	Zoologica Scripta 18(3): 405-410	4/20/4643
*Iberobathynella rouchi*	Camacho et Coineau	1987	Stygologia 3(2): 125-137	4/20/4641
*Iberobathynella imuniensis*	Camacho	1987	Archiv fur Hidrobiologia 111(1): 137-149	20.04/4642 & ADN/29166
***Hexaiberobathynella***	Camacho et Serban	1998	Travaux de l’Institut de Spéologie “É. Racovitza” 34: 15-75	
*Hexaiberobathynella hortezuelensis*	Camacho et Serban	1998	Travaux de l’Institut de Spéologie “É. Racovitza” 34: 15-75	4/20/4451
***Paraiberobathynella***	Camacho et Serban	1998	Travaux de l’Institut de Spéologie “É. Racovitza” 34: 15-75	
*Paraiberobathynella notenboomi*	(Camacho)	1989	Spixiana 12(2): 105-113	4/20/4644
***Guadalopebathynella***	Camacho et Serban	1998	Travaux de l’Institut de Spéologie “É. Racovitza” 34: 15-75	
*Guadalopebathynella puchi*	Camacho et Serban	1998	Travaux de l’Institut de Spéologie “É. Racovitza” 34: 15-75	4/20/4450
***Hexabathynella***	Schminke	1972		
*Hexabathynella sevillaensis*	Camacho	2005	Journal of Natural History 39(21): 1819-1838	20.04/5913 & ADN/29544-29545
*Hexabathynella valdecasasi*	Camacho	2004	Journal of Natural History 28: 1249-1261	4/20/4866
*Hexabathynella nicoleiana*	Camacho	1986	Bijdragen tot de Dierkunde 56(1): 123-131	20.04/4645 & ADN/29474
Bathynellidae				
***Vejdovskybathynella***	Serban et Leclerc	1984		
*Vejdovskybathynella vasconica*	Camacho, Dorda et Rey	2013	Graellsia 69(2): (in press)	20.04/9119-9141 & ADN/29623, 29633, 29635, 29638,29646
*Vejdovskybathynella edelweiss*	Camacho	2007	Journal of Natural History 41(45-48): 2817-2841	20.04/7791 & ADN/29413, 29469
*Vejdovskybathynella caroloi*	Camacho	2007	Journal of Natural History 41(45-48): 2817-2841	20.04/7792 & ADN/29877, 29897
*Vejdovskybathynella pascalis*	Camacho	2007	Journal of Natural History 41(45-48): 2817-2841	4/20/7793
***Paradoxiclamousella***	Camacho, Dorda et Rey	2013	Journal Natural History. 47 (21-22): 1393-1420	
*Paradoxiclamousella fideli*	Camacho, Dorda et Rey	2013	Journal Natural History. 47 (21-22): 1393-1420	20.04/8855-8876 & ADN/29746-29748 & 29750-29753
*Paradoxiclamousella pirata*	Camacho, Dorda et Rey	2013	Journal Natural History. 47 (21-22): 1393-1420	20.04/8877-8885 & ADN/29724, 29726-29727, 29911-29912

### Coordinates

36°17'24"N and 43°36'0"N Latitude; 9°49'12"W and 6°18'36"E Longitude

### Temporal coverage (specimens’ data range)

1949–2013

### Temporal coverage (collection formation)

1969-present

## Natural collections description

**Parent collection identifier:** NA

**Collection name:** Colección Camacho and Colección Crustaceos del MNCN

**Collection identifier:** http://www.gbif.es:8080/ipt/manage/resource.do?r=mncn-aic

**Specimen preservation method:** Ethanol 70%

**Curatorial unit:** 350 with an uncertainty of 0 (records)

## Methods

**Method step description:** The collection has been digitisated with MSEXCEL software, compatible with DarwinCorev 1.2 or Darwincore 1.4.

*Pre-digitisation phase*: The identifications of each specimen from each sample has been reviewed recently and some former imprecisions and the discovery of cryptic species (due for example to the use of molecular techniques) have lead modifying some records in the Excel file used as starting point for this work. The initial files were short on the number of fields for each of the sampling sites and dates of sampling (date, locality, province, habitat, collector and the species found with data on the family genus, species and author).

*Digitisation phase*: Starting from the initial Excel file, the standard fields for a DarwinCore v1.2 database were added as needed, and the geographical data was included (UTM coordinates) from a GPS in association to the samples taken (PASCALIS samples and all those taken after the year 2000), or were obtained from grey (speleological reports) or published (Notenboom and Meijers 1984; Puch 1998) literature (i.e., the precise location through GPS in the entrance of the caves where bathynellid samples have been collected), as well as from type specimens.

*Creation of the dataset*: The dataset was exported as a file in DarwinCore v1.2 format. DarwinCore elements included in dataset structure are listed in the dataset description section. A Darwin Core table was prepared from the original database project. The field-to-filed mapping was fine-tuned with the support of GBIF-Spain’s Coordination Unit. The resulted table was imported into the Darwin Test tool (http://www.gbif.es/darwin_test/Darwin_test_in.php, Ortega-Maqueda and Pando 2008). This tool allows detailed metadating of the dataset, and also performs a number of quality checks on the data (dataset structure compliance to Darwin core, geographic consistency, date format, etc. currently over sixty of those checks are carried out). Once the potential errors flagged have bee checked and corrected, a Darwin Core Archive is generated, also by the DarwinTest tool. The produced DwC-A is then uploaded to the GBIF-Spain’s IPT installation (http://www.gbif.es:8080/ipt/). From there, the dataset is made public, registered in GBIF and indexed and published by the GBIF data portal.

The dataset was transformed to a DarwinCore Archive format with metadata to ensure rapid discovery of this biodiversity resource and future publishing as a citable academic paper (see Chavan and Penev 2011)

**Figure 5.**
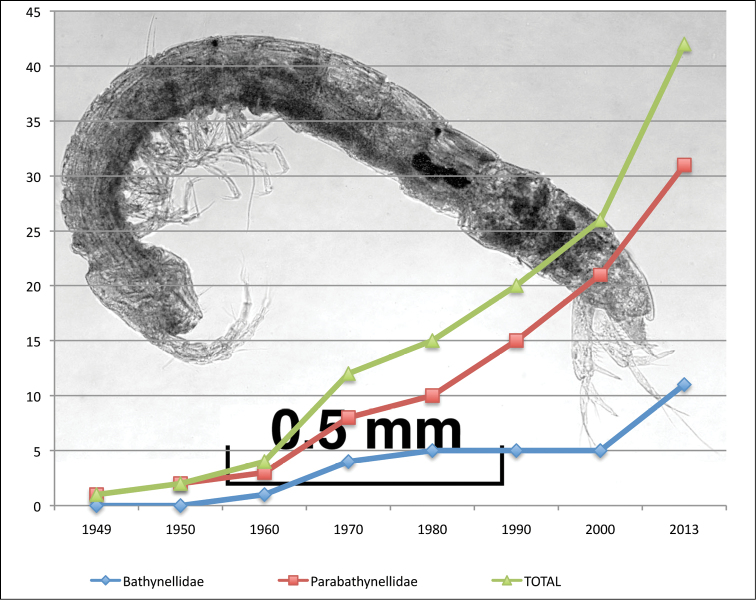
Cumulative curve distribution of knowledge on Bathynellacea since first discovery in Portugal until the present.

**Figure 6.**
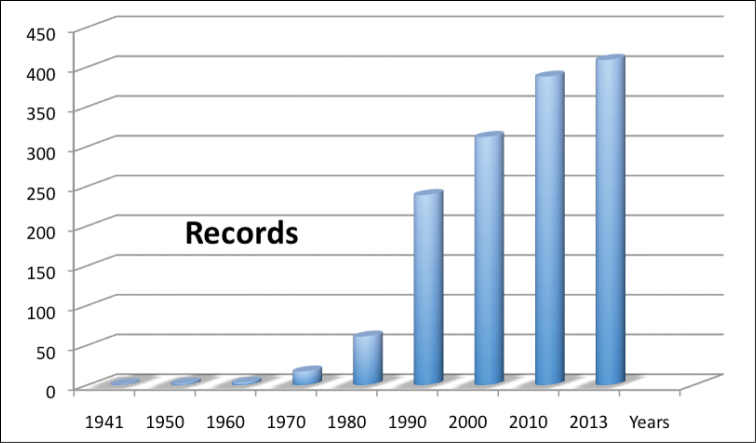
Distribution of records in the dataset by years.

**Study extent description:** This collection begins with the sampling campaigns by AIC in northern Spain for his doctoral thesis in 1983. Most of the data prior to 1976 are bibliographic (3.9%) although some samples studied by AIC were Bathyllenacea obtained between 1976 and 1978 by R. Rouch et coll. (8.3%), in three short sampling trips to different areas of the Iberian Peninsula. In addition, from 1984 to 1986 Jos Notenboom, assisted by Ines Meijers, and later P. van der Hurk & R. Leys (1986), took groundwater samples throughout Spain (12.7%) looking for stygobionts amphipods for the Notenboom doctoral Thesis and all Bathynellacea they found in these samples were also donated to AIC for study. The following years AIC has continued obtaining samples of this fauna throughout Spain in the framework of different research projects. It is worth noting the PASCALIS European project (2002–2004) (7.6%) in which AIC and his team conducted intensive sampling of groundwater fauna in the Cantabrian mountain ranges, an area where continuous sampling has been done since then together with C. Puch (65.3% of samples), increasing substantially the number of Bathynellacea records in Spain. The samples are mainly from the north of the Iberian Peninsula, Asturias, Cantabria and the north of Burgos (see Table 2 and Figures 1 and 3) although there is also a good representation of all the karstic areas of the Peninsula. The karstic areas of the Balearic Islands are still underrepresented (see grographic coverage section). The first sample recorded is from Portugal and was collected in 1949; the first bathynell from Spain dates from 1950 and is recorded for the Cueva de Genova (Genova cave) in Mallorca by the Romanian researchers Orghidan and Tabaccaru (Margalef 1951). Between the 50s and the 60s bathynells are found occasionally in samples from Portugal, Andalucia and Mallorca; in the 70s there are also few discoveries, but it is not until the 80s and from then on when most of the Bathynellacea samples of this dataset are found and studied. Figure 5 shows a graph of how the knowledge on bathynells has evolved along the last 70 years. Figure 6 shows the sampling efforts used in the Iberian Peninsula, translated into the number of records of bathynells included.The collection currently consists of over 409 samples with several thousand specimens and more than 2000 scientific preparations among which the type series of all new species described are included. The specimens are deposited in both the Collection of Crustaceans and the Tissues and DNA Collection of the MNCN.

**Sampling description:** Material of this collection has been collected in four ways:

1. Samples collected by Rouch et coll., in two short sampling campaigns in the Iberian Peninsula (1976 and 1977), which have been studied by AIC.
2. Samples collected in the sampling campaigns of Jos Notenboom et coll., in 1984, 1985 and 1986 to the Iberian Peninsula within the framework of his PhD thesis. These samples have also been studied by AIC.
3. Samples collected by AIC in 1983 for her PhD thesis (1987), plus samplings done in the framework of several research projects already mentioned, always with the collaboration of C. Puch and other speleologists (F. Molinero, A.M. de Juan, J. Robador, F. Lazaro, J. Bedoya).
4. Samples collected by AIC and her team as Spanish partner of the European Project “PASCALIS” (Cornisa Cantabrica) (2002–2004).

In addition some particular samples, with a more or less extense associated information, have been donated to AIC by fellow researchers (D.Jaume, A. Tinaut, J. Rodriguez, A. García-Valdecasas, P. Rodriguez, C. Boutin, E. Bello and C. Noreña).

The methods used in collecting this type of samples can be seen in Camacho 1992 and 1994. The samples are fixed in the field in formalin 4% or ethanol 96°, or are frozen. Each sample collected is studied under a binocular microscope in order to isolate the bathynellids specimens found.

The specimens used for morphological study are stored in alcohol (70%). The specimens used for molecular study are directly frozen at -80 °C. A complete dissection of all anatomical parts of specimens of type series is necessary for taxonomic study. The permanent preparations include the dissections together with entire specimens kept in special metal slides, using glycerine gelatine stained with methylene blue as the mounting medium. Anatomical examinations are performed using an oil immersion lens (100×) of an interference microscope.

The specific techniques used for molecular analysis for taxonomic application are detailed in Camacho et al. 2011, 2012 and 2013a.

**Quality control description:** Systematics reliability and consistency is backed by the experience of AIC, who made all identifications, in the field of Bathynellacea taxonomy. Recently, the identifications made are being confirmed by molecular data. The validation and cleaning of the associated geographical information has been introduced in several steps as a key issue of the digitisation process.

## Datasets

### Dataset description

**Object name:** Darwin Core Archive Iberian Peninsula and Balearic Island Bathynellacea (Crustacea, Syncarida) database

**Character encoding:** UTF-8

**Format name:** Darwin Core Archive format

**Format version:** 1.2

**Distribution:** http://www.gbif.es:8080/ipt/archive.do?r=mncn-aic

**Publication date of data:** 2013-09-24

**Update police:** Annually when necessary to transmit data of new samples o taxonomic changes.

**Language:** English

**Licenses of use:** This dataset [Iberian Peninsula and Balearic Island Bathynellacea (Crustacea, Syncarida) database] is made available under the Open Database License: http://opendatacommons.org/licenses/odbl/1.0/. Any rights in individual contents of the database are licensed under the Database Contents License: http://opendatacommons.org/licenses/dbcl/1.0/.

**Metadata language:** English

**Date of metadata creation:** 2013-09-10

**Hierarchy level:** Dataset
